# An Epidemic of 50 Cases of Influenza among the Personnel of a Base Hospital, B. E. F. France

**Published:** 1918-11

**Authors:** 


***An Epidemic of 50 cases of Influenza Among the Personnel of a Base Hospital, B. E. F. France.*** By C. J. MARTIN, *British Medical Journal,* Sept. 14, 1918.

*Epidemiology* : Twenty orderlies reported sick with fever, headache, and backache. During the following three days 5, 10 and 6 similar cases respectively occurred.

Three further cases occurred among the sisters during the next few days.

*Symptoms* : In every case there was headache, backache, and general malaise. The temperature reached 102° F. on the first or second evening, and in one case rose to 143° F. The duration of the fever was from 1 to 7 days.

Pain in the back of the eyes, and some tenderness of the globe was general. In 4 cases there was vomiting during the first day, and this was accompanied by diarrhea in one instance.

A feeling of rawness over the trachea was commonly experienced. An irritating cough occurred in 25 cases.

*Convalescence* : The temperature generally fell quickly to normal or nearly to normal, but it was not unusual for a subsequent rise to 99° to 100° F. to occur next day for a few hours, after which it remained normal. In cases in which the fever lasted for 5 days or upwards, convalescence was delayed.

*Bacteriological Investigation* : (*a*) Blood Culture.

Ten cc. of blood was drawn into 70 cc. citrated broth during the acute stage of the illness, (first or second day) from 24 patients. Apart from a few instances in which contamination had occurred, the flasks remained sterile.

(*b*) Investigation of Sputa.

Although many cases had a worrying cough, the amount of expectoration that came from the trachea was minimal. It was generally thin, clear mucus, full of bubbles, and its contents indicated that most of it was derived from the back of the throat. It was full of microbes of all sorts, and the author rarely succeeded in recovering anything resembling influenza bacillus from it.

(1) Observations upon Stained Films.

In the tracheal mucus from 8 patients, during the first or second day of the disease, occasional groups of Gram-negative bacilli resembling B. Influenzae were seen. And in similar material from 3 patients these were present in considerable numbers. As the sputum later became increasingly purulent, the influenza-like bacilli increased greatly in number and in proportion to other microbes. The bacilli were pleomorphic.

(2) Cultivation upon Blood Agar.

Usually the total number of colonies which grew upon blood agar plates, implanted from a washed piece of tracheal mucus, was small. From the sputum of 14 patients, taken at some period of the disease, minute colonies or small Gram-negative bacilli were obtained. In 5 instances such colonies were very abundant.

The bacilli were larger in cultures than they were in the original sputum, varying in dimensions from 1.5 μ by 0.5 μ to 0.6 μ by 0.4 μ. Some colonies exhibited some involution forms, usually 5 μ to 6 μ in length, but sometimes as long as 20 μ.

*Persistance of the Influenza-like Bacilli in the Respiratory Passages* : From one case, sputum teeming with organisms indistinguishable from Bacillus influenzae Pfeiffer was ejected each day.

For the first ten days the organisms were not seen inside the leucocytes, but from that date onwards the numbers of polymorphonucleated leucocytes, which contained these, rapidly increased. Coincident with this increase in phagocytic activity a marked improvement was noticed in the patient's condition.

In two cases phagocytosis of the bacilli was not observed until the 12th and 15th day respectively, and was coincident with a marked improvement in the pathological condition.

On the serum-glucose-agar plate of a 4th case a number of colonies grew which possessed all the characters of bacillus influenzae. In this case they had grown direct from the throat mucus without the assistance of blood, but in sub-culture they only grew from blood-agar. The bacilli were still present in the nasopharynx two weeks later.

*Leucocyte Count* : Total and differential enumeration of the leucocytes in the blood was made in 5 cases. The total number varied from 8000 to 12000 per cc., and the distribution of the different varieties was within normal limits.

*Agglutination of Patient's Serum* : By naked eye method, the highest agglutination titre reached was only 1 in 40 and in two controlled serums was 1 in 20. There was not a significant increase — a result which, the author believes, is consistent with previous experience of influenza.

